# Evaluation of gender determination using lip prints as an adjunctive tool among Indians

**DOI:** 10.6026/9732063002001215

**Published:** 2024-10-31

**Authors:** Manoj Meena, Monika Sharma, Bireswar Roy, Prasad Deeliprao Adhapure, Yogesh bande, Jayant Ambulgekar

**Affiliations:** 1 Department of Oral Medicine and Radiology, Rajasthan Dental College and Hospital, Jaipur, Rajasthan, India; 2 Department of Dentistry, Ananta Institute of Medical Sciences and Research Institute, Udaipur, Rajasthan, India; 3 Department of Oral Pathology and Microbiology, Sudha Rustagi College of Dental Sciences and Research, Faridabad, Haryana, India; 4 Department of Prosthodontics, CSMSS Dental College and Hospital, Kanchanwadi, Chhatrapati, Sambhajinagar, Maharashtra, India; 5 Department of Pediatric dentistry, Nanded Rural Dental College and Research Centre, Maharashtra, India; 6 Consultant Periodontologist, Ambulgekar's Smilax Dental Clinic, Chhatrapati, Sambhajinagar , Maharashtra, India

**Keywords:** Cheiloscopy, gender determination, lip prints

## Abstract

Cheiloscopy is defined as the study of lip prints. Lip prints are permanent and unique to each person and are useful in personal identification. Variations in lip prints patterns among males and females could help in sex determination to throw some light on lip prints and gender determination. The study was conducted on 100 subjects including 50 males and 50 females. Lip prints pattern were recorded and classified as per Tsuchihashi's classification into 6 types. Once the classification was done, then they were compared with each other for determining the uniqueness. The results obtained subjected to the statistical analysis. Majority of the subjects belonged to TYPE 1A and TYPE 1B. Type 1A and Type 1B (32%) was common in females and Type 1A (31%) was more common in males which was statistically significant in determining the gender (p value < 0.05). Lip prints hold the potential to identify gender determination.

## Background:

To establish a person's identity can be a very difficult process [1]. Dental, fingerprint and DNA comparisons are probably the most common techniques used in the forensic dentistry [2]. With the ever-increasing demands placed upon law enforcement to provide sufficient physical evidence linking a perpetrator to a crime, it makes sense to utilize any types of physical characteristic to identify a suspect of an offense. There are many well-known implanted methods of human identification, one of the most interesting emerging methods of human identification which originates from the criminal and forensic practice is human lip recognition [3]. Lip prints are the normal lines and fissures in the zone of transition of human lip between mucosa and the skin [4]. The study of lip prints is known as cheiloscopy [5]. Lip prints are permanent and unique to each person and are identifiable as early from 6th week of intrauterine life. They are useful in personal identification [6]. Apart from identifying and incidental use, lip prints can also be used as an important aid in sex determination based on its pattern. The grooves in the lip occur as distinct patterns or types and are unique to each individual and thus can be used to fix the identity of a person [7]. This biological phenomenon first noted by anthropologist R. Fischer [6]. Lip prints are unique as finger prints and do not change during the life of a person. It has been verified that they recover after undergoing alterations like trauma, inflammation and diseases like herpes and form of furrows does not vary with environmental factors [8, 9]. Variations in patterns among males and females could help in sex determination [7]. Therefore, it is of interest to evaluate gender determination using lip prints as an adjunctive tool among Indians.

## Methodology:

The study was conducted on 100 subjects including 50 males and 50 females in the age group18-23years were chosen randomly from undergraduate students of Bangalore Institute of dental sciences, Bangalore. Students whose lips were free from pathology and had normal transition zone between the mucosa and the skin were included whereas Students with gross deformities of lips (cleft lip, ulcers and traumatic injuries of lips) and who were allergic to lipstick were excluded. Lip prints pattern were recorded and classified as per Tsuchihashi's classification into 6 types [10]. To obtain the lip prints of the subjects, a dark colour lip stick was applied on each lip evenly using a lip stick applicator. A sheet of bond paper was folded and the hinged portion of the paper was inserted in between the lips and subjects were asked to press their lips onto it. It was then unfolded again. The lip prints obtained were observed using a Magnifying glass (10X) the lip print pattern of the middle part of the lower lip was considered for classification, as it is visible in almost all lip prints. Once the classification was done, then they were compared with each other for determining the uniqueness. The results obtained subjected to the statistical analysis.

## Results and Discussion:

Crimes always challenge the society in detection, diagnosis and identification of criminals. Identification of a person's identity can be a very difficult process. Human lips recognition is one of the most important methods of identification. Lip prints can be obtained at the crime scene from clothing, cups, glasses, cigarettes, windows and doors [11]. Remya *et al.* described in his study majority of his study belonged to type 4(26%). In males the predominant type was type 4 and females the predominant type was type 2 [12]. In another study conducted by Neeraj *et al.* in 128 subjects it was observed that type 1 pattern was predominant in both males and females [4]. In another study conducted by Bharathi *et al.* revealed in their study that most predominant lip print in the males was type 2 and females was Type 1B [6]. In a present study of 100 subjects (Table 1), it was observed that majority of the subjects belonged to TYPE 1A and TYPE 1B (32%). Data shows that Type 1A and Type 1B were common in females; Type 1A was common in males. The results were statistically significant in determining sex of the individual (p<0.05).

## Conclusion:

It was observed that majority of the subjects belonged to TYPE 1A and TYPE 1B. TYPE 4 (24%) is ranking next. Type 2, Type 3, Type 4 was the least types (4%) present at the group. Type 1A and Type 1B (32%) was common in females and Type 1A (31%) was more common in males which was statistically significant in determining the gender (p value < 0.05). Our present study also proved that lip prints hold the potential to identify gender determination. Further studies are recommended concerning the development of biometric system with more number of sample sizes to allow fast and more accurate assessment of the lip print.

## Figures and Tables

**Table 1:** Showing Lip prints matching for gender determination.

	**Matched types MALES**	**Percent**	**p value Chi-square**	**Matched types FEMALES**	**Percent**	**p value Chi-square**
TYPE 1A	8	32	.038*	9	31%	.003*
TYPE 1B	8	32	.001*	9	31%	0.587
TYPE 2	1	4	0.135	2	6.80%	0.659
TYPE 3	1	4	0.565	0	0	0.386
TYPE 4	6	24	0.135	2	6.80%	0.487
TYPE 5	1	4	0.25	7	24%	0.157
TOTAL	25	100	0.00*	29	100	.000*
